# Anti-fibrotic properties of an adiponectin paralog protein, C1q/TNF-related protein 6 (CTRP6), in diffuse gastric adenocarcinoma

**DOI:** 10.7150/jca.46765

**Published:** 2021-01-01

**Authors:** Yoshinori Iwata, Itaru Yasufuku, Chiemi Saigo, Yusuke Kito, Tamotsu Takeuchi, Kazuhiro Yoshida

**Affiliations:** 1 Department of Surgical Oncology, Gifu University, Gifu University Graduate School of Medicine, Gifu, Japan.; 2 Department of Pathology and Translational Research, Gifu University Graduate School of Medicine, Gifu, Japan.

**Keywords:** CTRP6, diffuse-type gastric cancer, cancer stromal microenvironment, myofibroblast, desmoplastic reaction

## Abstract

Patients with advanced gastric cancer, especially diffuse-type gastric cancer, which is often accompanied by stromal fibrosis, commonly exhibit a poor prognosis. This study was designed to unravel the potential roles of C1q/TNF-related protein 6 (CTRP6) in the fibrotic cancer microenvironment of diffuse-type gastric adenocarcinoma. A total of 49 diffuse-type gastric cancer samples were evaluated in this study, and 23 of these samples exhibited focal CTRP6 immunoreactivity. CTRP6 immunoreactivity was found to be correlated with favorable survival outcomes, in terms of both overall and relapse-free survival rates, but this trend did not reach significance (*P* = 0.15). By contrast, CTRP6 immunoreactivity was significantly correlated with relapse-free survival rates in patients with diffuse-type gastric cancer at a distal site (*P* = 0.028). Notably, most gastric cancer cells at the cancer invasive front were CTRP6 negative, especially in areas of robust fibrosis. Double immunohistochemical staining demonstrated an inverse expression profile for CTRP6 and the activated fibroblast marker alpha smooth muscle actin (α-sma) in stromal and gastric cancer cells at the cancer invasion front. The addition of recombinant CTRP6 protein attenuated the TGF-β-induced α-sma expression in cultured human fibroblasts but did not alter the proliferation rate or Matrigel-invasion activity of the cultured gastric cancer cells. In addition, CTRP6 did not affect the viability of normal human gastric epithelial cells. This study suggests that CTRP6 may have potential application in combating stromal fibrosis in diffuse-type gastric cancers.

## Introduction

Gastric cancer remains one of the most prolific cancers worldwide 1. Although gastric cancer is characterized by highly heterogeneous tumors, it can generally be classified into two types, intestinal and diffuse according to the Lauren classification system 2. Despite advances in treatment, the prognosis of patients with advanced gastric cancer, especially diffuse-type gastric cancers, which are often characterized by robust fibrosis, remains poor 3. Cancer-associated fibroblasts facilitate its progression by secreting a variety of soluble factors via direct cancer-stromal interactions. Therefore, cancer stromal fibrosis could be a novel target in the treatment of patients with various malignant tumors, including gastric cancer 4, 5.

Recent studies have described an inhibitory effect mediated by the adiponectin paralog protein CTRP6 [C1q/TNF-related protein 6, also known as C1qTNF6; gene symbol *CTRP6*] on inflammation 6 and inflammation-related fibrosis in several organs, including cardiac fibrosis 7, dermal fibrosis 8, and renal fibrosis 9. However, it was unclear whether CTRP6 would have any anti-fibrotic activity in the cancer stroma.

In this study, we aimed to examine the effects of CTRP6 expression on cancer stromal fibrosis in diffuse-type gastric cancer. Our findings indicate that CTRP6 may have an antagonistic effect on cancer stromal fibrosis in diffuse-type gastric cancer.

## Materials and methods

### Patients

After receiving the approval of the Institutional Review Board of the Gifu University Graduate School of Medicine (specific approval numbers: 2019-202) to carry out our retrospective study, we collected consecutive 49 specimens from surgically treated patients between January 2005 and December 2011, at Gifu University Hospital, who were primarily diagnosed with diffuse-type gastric cancer. Informed consent was obtained from all participants or their authorized representatives. This study was conducted in accordance with the ethical standards outlined in the 1975 Helsinki Declaration.

### Immunohistochemical staining

All tissue specimens were fixed in 10% buffered formalin and embedded in paraffin. The tissues were immune-stained with antibodies conjugated to the ImmPRESS™ polymerized reporter enzyme staining system (Vector Laboratories, Burlingame, CA, USA) as previously described 10. Rabbit specific antibody to CTRP6 was purchased from Atlas Antibodies AB (product No. HPA002042) (Stockholm, Sweden). Monoclonal antibody against α-smooth muscle actin (α-sma) (clone 1A4) was purchased from Dako Agilent (Santa Clara, CA). Double immunohistochemical staining was performed as previously reported 11.

### Evaluation of immunohistochemical staining and statistical analysis

We expressed the immunohistochemical staining results as a percentage, calculated from the proportion of immunoreactive gastric adenocarcinoma cells relative to the total number of cells. The proportion of positive cells was determined by scoring 10 high-magnification fields of view (400× through the most superficial to the deepest layers of the main invasion areas) for each sample. The staining was considered negative if less than 10% of the cancer cells exhibited immunoreactivity and positive if over 10% were immunoreactive.

### Cells and cell culture

In this study, we used five gastric cancer cell lines. The KATO III 12, MKN7 13, and MKN74 13 gastric cancer cell lines were obtained from the Riken Cell Bank (Tsukuba, Japan). The NUGC-4 14 gastric cancer cell line was obtained from the Japanese Cell Research Bank (Osaka, Japan). GPM-2 gastric cancer cells 15 were a kind gift from Dr. Hayao Nakanishi (Division of Oncological Pathology, Aichi Cancer Center Research Institute), who established this cell line. KATO III, NUGC-4, and GPM-2 cells are diffuse-type gastric cancer cells, whereas MKN7 and MKN74 cells are intestinal-type gastric cancer cells. Cells were cultured in Dulbecco's modified Eagle's medium (Gibco Life Technologies, Grand Island, NY) containing 10% heat-inactivated fetal bovine serum without any antibiotics. Cells were passaged for no more than 6 months after resuscitation.

Human gastric epithelial cells, designated as HGaEpC were purchased from Cell Applications (San Diego, CA, USA). HGaEpC cells (lot 3288) were cultured with manufacturer-supplied medium.

Sf21 cells were cultured in Grace's Insect Medium (Gibco Life Technologies) containing 10% FBS and used to prepare the P3 supernatants. To obtain the recombinant protein, Sf21 cells were cultured in serum-free Sf-900™ III SFM culture medium (Gibco Life Technologies).

Adult human dermal fibroblast cells were purchased from Thermo Fisher Scientific, Inc. (Waltham, MA) and cultured in Medium 106 supplemented with Low Serum Growth Supplement (Thermo Fisher Scientific).

### Recombinant CTRP6 protein and TGF-β

Recombinant CTRP6 was generated using a Bac-to Bac Baculovirus expression system (Invitrogen Co, San Diego, CA) as previously reported 16. Briefly, cDNA encoding full length human CTRP6 followed by a His-tag, was subcloned into a pFastBac1 transfer vector, verified by sequencing, and transfected into *E. coli* strain DH10Bac™, which contains a Baculovirus shuttle vector (bacmid) and a helper plasmid. These cells were used to generate a recombinant bacmid according to the manufacturers' protocol. Finally, P3 recombinant Baculovirus stock was prepared for generating recombinant CTRP6-His in Sf21 cells. Culture supernatant from infected Sf21 cells was added to an Ni-NTA Spin Kit (Qiagen, Valencia, CA) for purification. The concentration of purified recombinant protein, CTRP6-His, was measured using a BCA Protein Assay Kit (Takara, Ohtsu, Japan).

Recombinant human TGF-β1 was purchased from PeproTech, (Rocky Hill, NJ).

### Reverse transcription PCR (RT-PCR) and quantitative RT-PCR (qRT-PCR)

cDNA synthesis from total RNA and the subsequent PCR were performed using a Reverse Transcription Polymerase Chain Reaction (RT-PCR) Kit (TaKaRa, Shiga, Japan) as previously described 17. qRT-PCR was performed using a SYBR Green Reaction Kit according to the manufacturer's instructions (Roche Diagnostics, GmbH, Mannheim, Germany) on a LightCycler (Roche Diagnostics).

The sequences of the primers used in this study are as follows: for *CTRP6* forward, 5′-ATTCCTGCTTCCTCTTGTGTTT-3′, and reverse, 5′-GACAGCCTTTGGGGAGATG-3′; for *α-sma* forward, 5′- GACAATGGCTCTGGGCTCTGTAA -3′, and reverse, 5′- CTGTGCTTCGTCACCCACGTA -3′; and for *GAPDH* forward 5′-GAAGGTGAAGGTCGGAGTC-3′, and reverse 5′- GAAGATGGTGATGGGATTTC-3′.

The expression of each target gene was analyzed using the 2^-ΔΔCT^ method 18 embedded in the LightCycler system. ΔCT values for each gene of interest were normalized to the *GAPDH* values for each triplicate. Standard deviations were then calculated for each triplicate, and the fold change for each of the three target genes was recorded. The value for each of the groups (n = 3) was calculated as the fold change relative to the mean value for the control siRNA-treated group (control set to 1.0).

### Western blot

Western blot was carried out according to the method described by Towbin et al. 19 with modifications as previously described 17. Briefly, proteins were electrophoresed on sodium dodecyl sulfate polyacrylamide gel electrophoresis (SDS-PAGE) gels and electroblotted to polyvinylidene difluoride membranes (Millipore, Bedford, MA). The membranes were blocked with bovine serum albumin and subsequently incubated with rabbit anti-CTRP6 (Atlas Antibodies AB) or anti-α-SMA (Cat No. 55135-1-AP; Proteintech, IL) antibodies. Immunoblot bands were quantified by densitometry using LI-COR C-DiGit Blot Scanner imaging software version 3.1 (LI-COR Biosciences, Lincoln, NE, USA) and normalized against the GAPDH band as previously described 20.

### Immunofluorescence staining

Cells were fixed with 4% (m/v) paraformaldehyde, permeabilized with 0.1% TritonX-100, and blocked with 10% goat serum. Then the cells were incubated with 1 μg/mL rabbit anti-α-sma (Proteintech) antibody at room temperature for 1 h. After washing with PBS, cells were incubated with Alexa Fluor 488-conjugated anti-rabbit antibody (1:200) (Invitrogen, Carlsbad, CA). After staining, images were acquired using a confocal laser scanning microscope (Leica TCS SP8, Germany).

### Cell proliferation and Matrigel-invasion assays

Cell proliferation was evaluated by counting the number of viable cells as previously described 11. Briefly, 0.5 × 10^4^ cells were cultured in tissue culture dishes with or without recombinant CTRP6 in triplicate. After 24, 48, and 72 h, the viable cells were counted. The assay was conducted in triplicate and repeated twice.

The invasiveness of the cultured cells was determined using 24-well BD BioCoat Matrigel Invasion Chamber Plates (BD Falcon, Bedford, MA) according to the manufacturer's protocol as previously described 11. Briefly, 1 × 10^4^ cells were added to the upper compartment of the invasion chamber with or without recombinant CTRP6. After incubation for 24, 48, and 72 h in DMEM containing 10% (lower chamber) or 0% (upper chamber) FBS, non-invading cells were gently removed from the filter by scrubbing with a cotton-tipped swab. The cells on the lower surface of the filter were counted using a microscope. These experiments were performed in triplicate and repeated twice. The mean and standard deviation values were calculated.

### Statistical analysis

Curves for overall survival (OS) and relapse-free period (RFP) were drawn using the Kaplan-Meier method, and the differences in survival rates were compared using the log-rank test for univariate survival analysis. Multivariate Cox proportional hazard regression analysis was performed to calculate a hazard ratio of death for CTRP6.expression. For quantitative RT-CR, Western blot, cell proliferation, and matrigel-invasion assays, statistical analysis was performed by ANOVA using Tukey's test. *P* value of < 0.05 was considered statistically significant.

## Results

### CTRP6 expression in diffuse-type gastric adenocarcinoma tissue specimens and its relationship with prognosis

**Figure 1:**
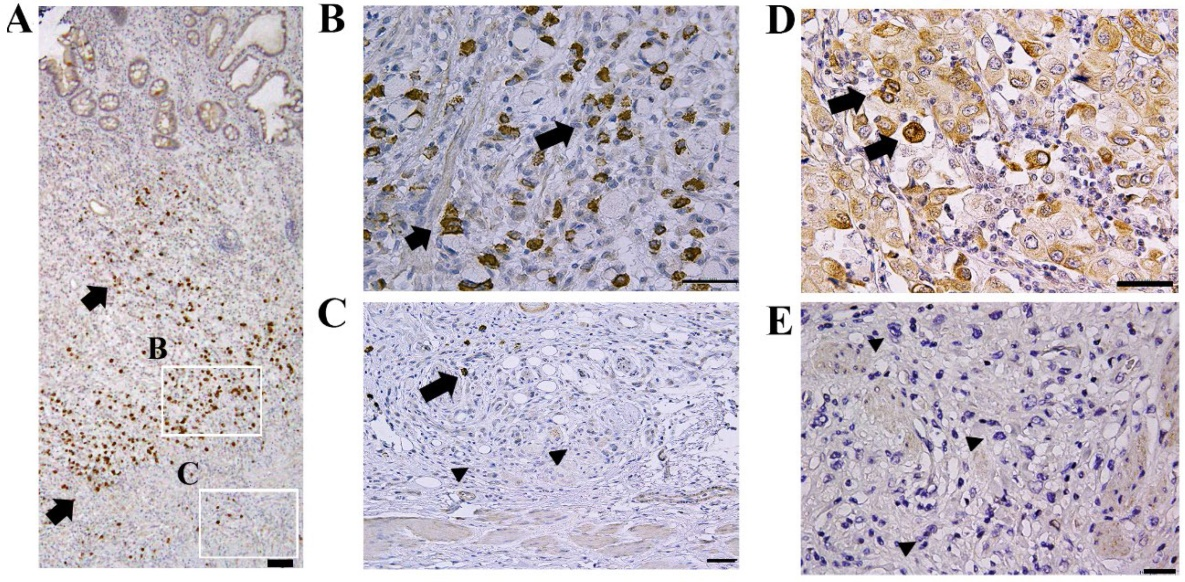
** Expression of CTRP6 in diffuse-type gastric cancer.** Gastric cancer cells exhibit CTRP6 immunoreactivity (A and B) but were CTRP6 negative at the cancer invasion front (C). CTRP6 immunoreactivity was also found in the lamina propria of areas with minimal fibrosis (D) but was not expressed at deeply invaded sites with robust fibrosis (E). Arrows indicate cancer cells with CTRP6 immunoreactivity, whereas arrow heads indicate CTRP6-negative cancer cells. Scale bar represents 50 µm.

Representative results of the immunohistochemical staining are shown in Fig. 1. Limited CTRP6 expression was observed in non-tumorous gastric epithelial cells. Although CTRP6 immunoreactivity was found focally in 23 of the 49 invasive cancer samples (Fig. 1A, B, and D), CTRP6 immunoreactivity was lost in the invasion front cells in the tissue specimens examined here (Fig. 1C and E).

**Figure 2:**
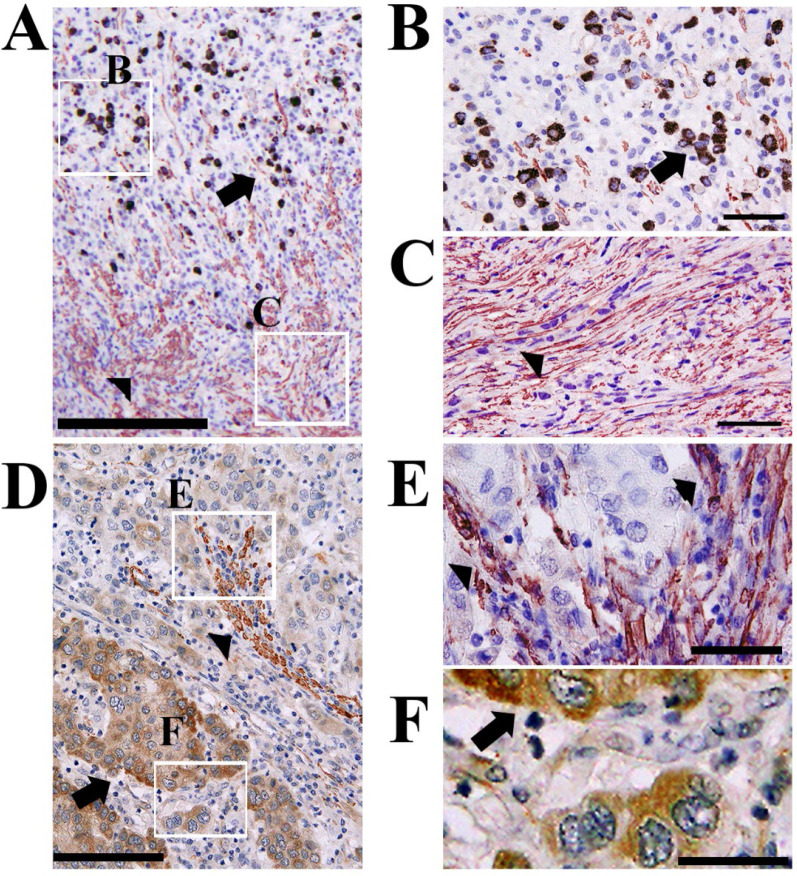
** Inverse expression patterns of CTRP6 and α-sma in cancer and surrounding stromal cells.** Double immunohistochemical staining showed that CTRP6 (Brown) was expressed in gastric cancer cells and α-sma (Red) was expressed in stromal cells with inverse expression profiles within the cancer microenvironment and at the cancer invasion front. Arrows and arrow heads indicate CTRP6-positive cancer cells and α-sma- positive stromal cells, respectively. Scale Bars indicate: 200 µm (A), 100 µm (D), and 50 µm (B, C, E, and F).

The relationship between CTRP6 immunoreactivity and clinicopathological factors, including OS and RFP rates, is shown in Table 1 and Fig 2. CTRP6 immunoreactivity was shown to be more common in diffuse-type gastric cancer patients who experience a favorable outcome, either improved OS or RFP rate, but this trend did not reach significance (*P* = 0.15). Multivariate analyses of risk factors for survival demonstrated that CTRP6 immunoreactivity was not independently related to prognosis. By contrast, CTRP6 immunoreactivity was significantly related to favorable RFP in patients with diffuse-type gastric cancer at a distal site (*P* = 0.028).

**Figure 3:**
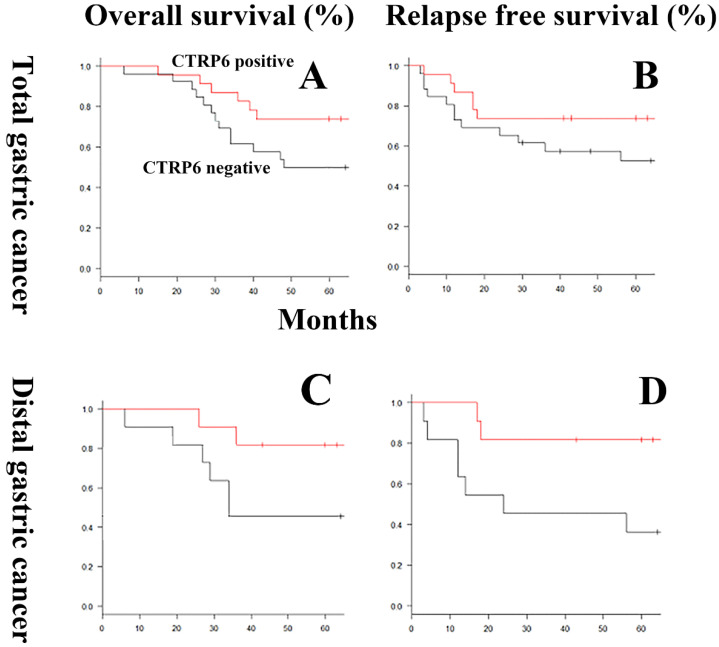
** Overall survival and relapse-free survival curves using CTRP6 immunoreactivity in diffuse-type gastric cancers.** In general, for the gastric cancers evaluated here, the overall (A) and relapse-free (B) survival rates of patients expressing CTRP6 was better than that of patients without CTRP6, but this trend did not reach significance (*P* = 0.15). In distal gastric cancer, CTRP6 immunoreactivity was not significantly correlated with prognosis (*P* = 0.07). By contrast, CTRP6 immunoreactivity did demonstrate a significant correlation with improved relapse-free survival rates in patients with distal gastric cancer (*P* = 0.028).

Double immunohistochemical staining demonstrated an inverse expression profile for CTRP6 expression in cancer cells and α-sma expression in surrounding stromal cells (Fig. 3).

### Recombinant CTRP6 significantly suppressed α-sma expression in cultured fibroblasts

**Figure 4:**
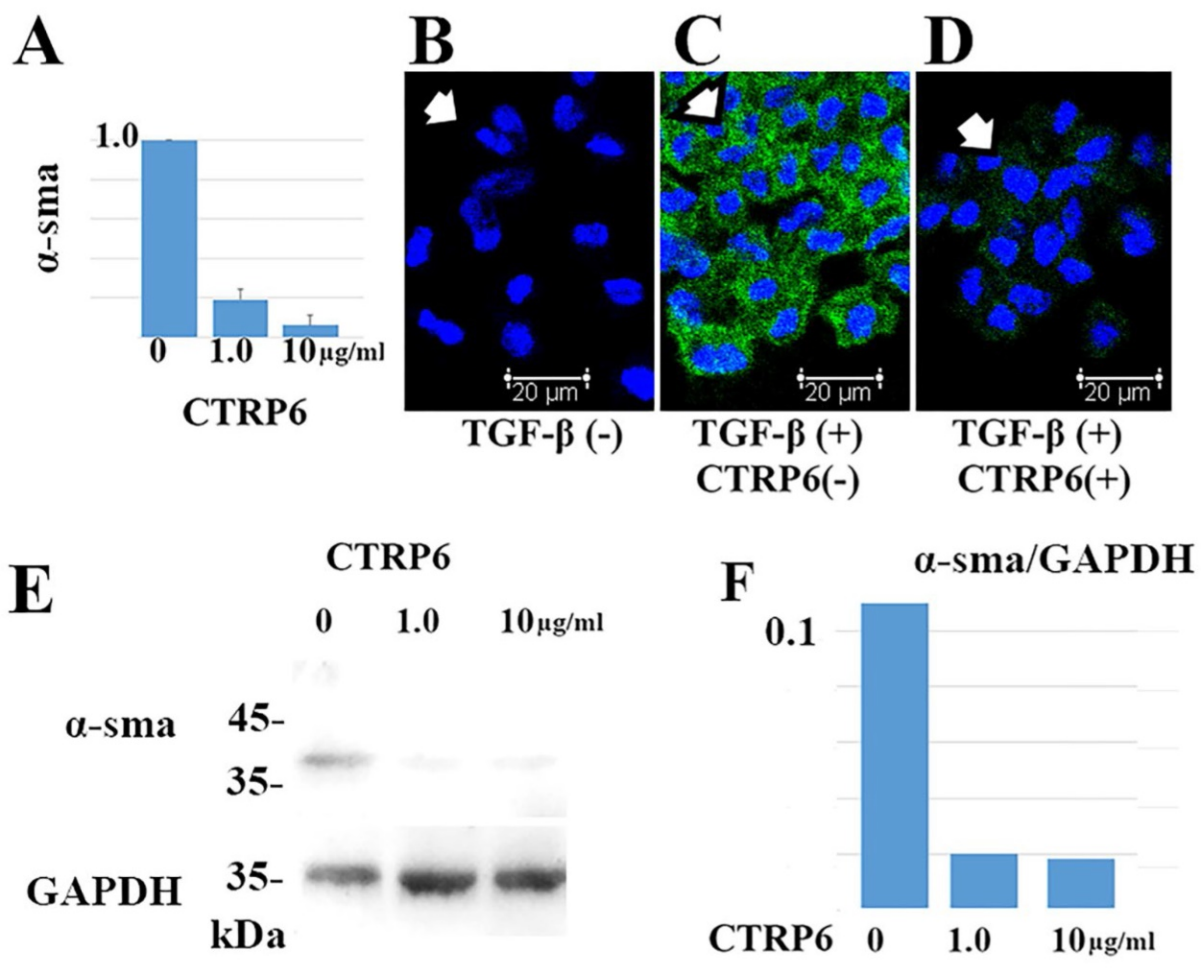
** CTRP6 significantly suppresses α-sma expression in cultured fibroblasts.** Administration of both 1.0 and 10 µg/mL CTRP6 significantly reduced α-sma expression at the transcript (A) and protein level (B-F) at 72 h (A and F: *P* < 0.01). (A) Representative data of the quantitative RT-PCR assay. The value for each group (n = 3) was calculated as the fold change relative to the mean value for the control group (control set to 1.0), which represents the *α-sma* transcription induced by TGF-β without CTRP6. (B-D) Immunofluorescence assays demonstrated that α-sma (green staining; Alexa Fluor 488) was expressed in the cytoplasm of fibroblasts (indicated by arrow) treated with TGF-β (C), but not found in fibroblasts without TGF-β (B). CTRP6 (1.0 µg/mL) attenuated TGF-β induced α-sma expression in cultured fibroblasts (D). Scale bar, 20 µm. (E and F) Immunoblots revealed an α-sma positive band when fibroblasts were treated with TGF-β. By contrast, α-sma band was observed to a lesser extent in fibroblasts that were treated with both TGF-β and CTRP6. The intensity of the GAPDH reference did not change. Semi quantitative analysis was performed as described in the Materials and methods. The intensity ratio of each band relative to GAPDH is shown. The CTRP6/GAPDH ratio was 0.11 (Mock), 0.02 (1.0 µg/mL CTRP6 treated group), and 0.018 (10 µg/mL CTRP6 treated group).

TGF-β promotes differentiation of fibroblasts into α-sma-positive myofibroblasts in cancer stroma. We asked whether CTRP6 could attenuate α-sma expression in TGF-β treated cultured fibroblasts. Representative results are shown in Fig 4. Notably, CTRP6 suppresses α-sma expression at both the transcript and protein level. This result indicates that CTRP6 could suppress TGF-β induced conversion of fibroblasts to myofibroblast *in vitro*.

### Recombinant CTRP6 did not alter the proliferation or Matrigel-invasion activity of gastric cancer cells

**Figure 5:**
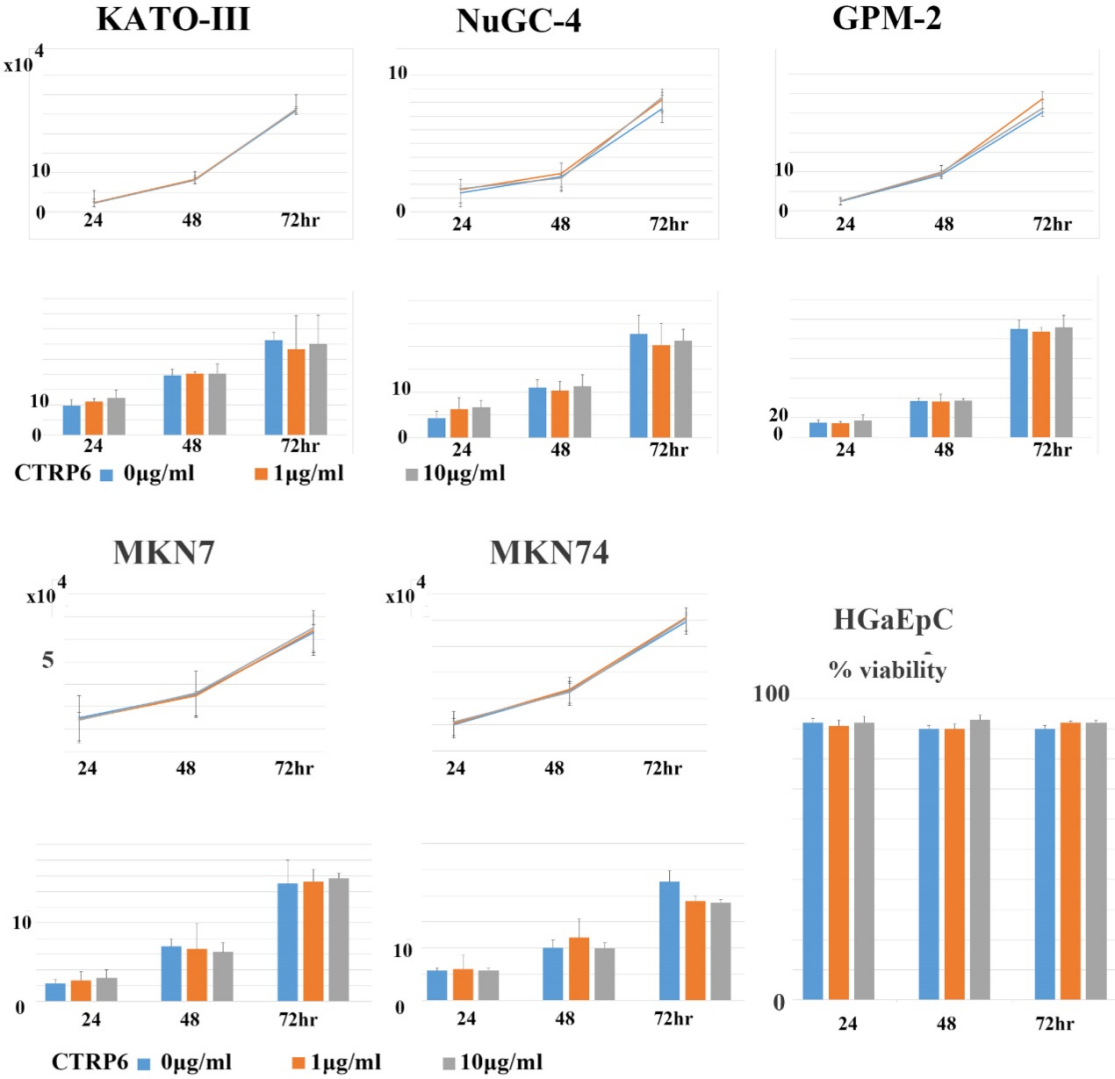
** CTRP6 expression did not alter the cell growth or Matrigel-invasion activities of gastric cancer cells or the viability of primary cultured gastric epithelial cells.** Upper lane: Addition of neither 1.0 nor 10 µg/mL of CTRP6 affected the cell growth parameters of three diffuse or two intestinal-type gastric cancer cells after 24, 48, and 72 hr of incubation. Lower lane: Addition of neither 1.0 nor 10 µg/mL of CTRP6 affected the Matrigel-invasion activity of gastric cancer cells at 24, 48, and 72hr of incubation. No significant difference was observed in cell growth and Matrigel-invasion assay. 1.0, nor 10 µg/mL of CTRP6, affected the cell viability of primary cultured gastric epithelial cells, HGaEpC after 24, 48, and 72 hr of incubation.

Subsequently, we asked whether CTRP6 expression could alter the proliferation or invasion activity of gastric cancer cells. Representative findings are shown in Fig. 5. We did not observe any significant alternation to proliferation or matrigel invasion in diffuse-type or intestinal-type gastric cancer cells when we treated the cells with recombinant CTRP6.

### Recombinant CTRP6 did not alter the cell viability of primary cultured normal gastric epithelial cells

Finally, we examined whether CTRP6 altered the cell viability of normal gastric epithelial cells. As shown in Fig. 5, we did not find any difference in the viability of normal primary cultured gastric epithelial cells.

## Discussion

Development of cancer stromal fibrosis is believed to promote cancer progression and confer chemotherapy resistance properties to malignant tissues 21. Expansion of these fibroblasts followed by their conversion to an active status, i.e., myofibroblasts, is critical in the development of cancer stromal fibrosis. Diffuse-type gastric cancers, including scirrhous carcinoma, are often accompanied by robust fibrosis in their advanced stages. In this study, we examined the effect of CTRP6 expression on diffuse gastric cancer focusing on cancer stromal fibrosis. The double immunohistochemical staining demonstrated that α-sma was expressed in cancer stroma whereas CTRP6 was expressed in cancer cells, and there was limited overlap in the expression profiles of these two proteins (Fig. 3). The addition of recombinant CTRP6 significantly reduced the TGF-β-induced differentiation of fibroblasts into α-sma-positive myofibroblasts (Fig. 4). Since the expression of α-sma is commonly used to identify activated mesenchymal cells at the sites of desmoplastic cancer stroma, we believe that CTRP6 expression may also have an inhibitory effect on cancer stromal fibrosis in gastric cancer, which is consistent with reports of its expression in post infarct cardiac fibrosis 7, dermal fibrosis 8, and renal fibrosis 9.

According to Lauren's criteria, gastric cancer is classified into two main types: Intestinal and diffuse 2. Previously, Qu et al. reported that CTRP6‑knockdown decreased the growth of an AGS cell line 22 that is commonly used to study intestinal type gastric cancer phenotypes 23. By contrast, this study demonstrated that the addition of recombinant CTRP6 did not alter the cell growth or Matrigel-invasion activity of diffuse-type gastric cancer cells.

Combined with the findings of Qu et al. 22, elimination of endogenous CTRP6 may abrogate the progression of gastric cancer, whereas exogenous overload of CTRP6 might have little effect on non-tumorous or cancerous gastric epithelial cells themselves. Furthermore, we found that CTRP6 immunoreactivity correlated with favorable survival outcomes, in terms of both overall and relapse-free survival rates, but this trend did not reach significance (*P* = 0.15). This may reflect the pleiotropic pathobiological property of CTRP6 in gastric cancer cells, themselves, and in cancer-stromal microenvironments. This observation requires further extensive studies that aim to elucidate the pathobiological properties of CTRP6 in gastric cancer cells.

In this study, CTRP6 expression was not significantly related to the prognosis of patients with whole diffuse-type gastric cancer. By contrast, CTRP6 immunoreactivity was significantly correlated with positive prognosis in patients with diffuse-type gastric cancer at distal sites. A recent national cancer data base study, which was comprised 97,060 patients with gastric cancer clearly indicated that proximal and distal gastric cancers differ in their clinicopathologic characteristics, including prognosis 24. Fan et al. also suggested that heterogeneity between proximal and distal gastric cancers may be related to differences in their tumor biology 25. Although the difference in the prognostic value of CTRP6 expression between proximal and distal gastric cancer may be the result of the inherent pathobiological heterogeneity of proximal and distal gastric cancers, further extensive studies are needed for verification.

Notably, recent independent cohorts that are publicly available in Kaplan-Meier plotter database (http://kmplot.com/analysis/index.php?p=service&cancer=gastric) reveal that “high *CTRP6* mRNA expression” is more common in patients who experience a favorable outcome, although this trend does not reach statistical significance (*P* = 0.33). The patients who were included in this analysis were diagnosed with diffuse-type and poorly differentiated gastric cancer and were treated by surgical resection. However, information concerning cancer location, i.e. proximal or distal site, is not available in this database.

The IntAct (https://www.ebi.ac.uk/intact/) interaction database suggests a putative interaction between CTRP6 and protein of human promyelocytic leukemia (PML), based on an anti-tag coimmunoprecipitation assay. The BioGrid interaction database (https://thebiogrid.org/interaction) also indicates an interaction between CTRP6 and PML protein, through yeast-two hybrid screening. Recent studies have demonstrated that PML protein plays an important role in fibrosis, i.e. TGF β-induced fibroblast-to-myofibroblast differentiation 26, 27. Although this finding needs to be verified through further extensive studies, we hypothesize that CTRP6 may attenuate PML protein-related fibrosis in cancer-stromal microenvironments.

In conclusion, this study revealed that CTRP6 may act as an antagonist to cancer stromal fibrosis in diffuse-type gastric cancer at the cancer invasion front.

## Figures and Tables

**Table 1:** Correlation between CTRP6 immunoreactivity and pathological features

Characteristics	CTRP6-positive (n=23)	CTRP6-negative (n=26)	*P*-value
**Age (years)**			
Mean (range)	61.0 (32-87)	64.2 (31-84)	
< 65	13 (57)	13 (50)	0.43
≥ 65	10 (43)	13 (50)	0.65
**Sex**			0.40
Male	10 (43)	15 (58)	
Female	13 (57)	11 (42)	
**Tumor location**			0.78
Lower third	11 (48)	11 (42)	
Upper or middle third	12 (52)	15 (58)	
**Component of Signet ring cell**			0.56
Positive	9 (39)	8 (31)	
Negative	14 (61)	18 (69)	
**pT**			0.064
T1-2	7 (30)	2 (8)	
T3-4	16 (70)	24 (92)	
**pN**			1
N0	5 (22)	6 (23)	
N1-3	18 (78)	20 (77)	

**Table 2:** Univariate and Multivariate analyses of risk factors for survival

Characteristics	Univariate			Multivariate		
	HR	95% CI	*P*-value	HR	95% CI	*P*-value
**Age**						
< 65	1					
≥ 65	0.919	0.370-2.223	0.85			
**Sex**						
Male	1					
Female	0.800	0.321-1.934	0.62			
**Tumor location**						
Lower third	1					
Upper or middle third	1.147	0.474-2.928	0.76			
**Component of Signet ring cell**						
Positive	1					
Negative	0.953	0.394-2.437	0.92			
**pT**						
T1-2	1			1		
T3-4	2.06e+9	3.054-3.054	0.0011	1.46e+9	1.946-none	0.0096
**pN**						
N0	1			1		
N1-3	3.102	0.893-19.54	0.079	1.787	0.513-11.27	0.40
**CTRP6**						
Positive	1			1		
Negative	1.946	0.796-5.183	0.15	1.441	0.589-3.843	0.43

## Abbreviations

CTRP6: C1q/TNF-related protein 6
α-sma: α-smooth muscle actin
SDS-PAGE: sodium dodecyl sulfate polyacrylamide gel electrophoresis
OS: overall survival
RFP: relapse-free period
